# Impacts of Solid-State Fermented Barley with Fibrolytic Exogenous Enzymes on Feed Utilization, and Antioxidant Status of Broiler Chickens

**DOI:** 10.3390/vetsci10100594

**Published:** 2023-09-27

**Authors:** Doaa Ibrahim, Hassainen I. El-sayed, Elsabbagh R. Mahmoud, Ghada I. Abd El-Rahman, Shefaa M. Bazeed, Abdelwahab A. Abdelwarith, Aya Elgamal, Samah S. Khalil, Elsayed M. Younis, Asmaa T. Y. Kishawy, Simon J. Davies, Abdallah E. Metwally

**Affiliations:** 1 Department of Nutrition and Clinical Nutrition, Faculty of Veterinary Medicine, Zagazig University, Zagazig 44519, Egypt; 2 Department of Clinical Pathology, Faculty of Veterinary Medicine, Zagazig University, Zagazig 44519, Egypt; gana660@gmail.com; 3 Department of Biochemistry and Chemistry of Nutrition, Faculty of Veterinary Medicine, Badr University in Cairo (BUC), Cairo P.O. Box 4942301, Egypt; shefabazeed@gmail.com; 4 Department of Zoology, College of Science, King Saud University, P.O. Box 2455, Riyadh 11451, Saudi Arabia; awarith@ksu.edu.sa (A.A.A.); emyounis@ksu.edu.sa (E.M.Y.); 5 Department of Animal Histology and Anatomy, Faculty of Veterinary Medicine, Badr University in Cairo (BUC), Cairo P.O. Box 4942301, Egypt; doctorayaelgamal@gmail.com; 6 Department of biochemistry, drug information center, Zagazig University Hospitals, Zagazig University, Zagazig P.O. Box 44511, Egypt; samahsaid75@gmail.com; 7 Aquaculture Nutrition Research Unit ANRU, Carna Research Station, Ryan Institute, College of Science and Engineering, University of Galway, H91 V8Y1 Galway, Ireland; sjdplymouth@live.co.uk

**Keywords:** solid state fermentation, barley grains, fibrolytic enzymes, broiler chickens, intestinal barriers, nutrient transporters

## Abstract

**Simple Summary:**

The efficient feed utilization of raw feed ingredients is one of the main factors associated with superior growth and production in poultry farming. The higher demand for cereal grains as energy sources has encouraged the dietary inclusion of other alternative cereals to achieve the target poultry production. However, alternative cereals such as barley grains may limit poultry growth due to their higher content of anti-nutritional factors, such as non-starch polysaccharides (NSPs). Hence, the application of solid-state fermentation technology with fibrolytic enzymes allows for a higher dietary inclusion of barley comparable to its actual inclusion levels. In this study, including 10% fermented and enzymatically treated barley not only improved feed utilization efficiency, but also modified intestinal barrier functions and antioxidant status and upregulated the expression of nutrient-transport-related genes. Therefore, fermented and enzymatically treated barley can be used as a promising alternative to corn and achieve the target production of broiler chickens.

**Abstract:**

The present and future high demand of common cereals as corn and wheat encourage the development of feed processing technology that allows for the dietary inclusion of other cereals of low nutritional value in poultry feeding. Barley grains contain anti-nutritional factors that limit their dietary inclusion in the poultry industry. The treatment of barley with solid-state fermentation and exogenous enzymes (FBEs) provides a good alternative to common cereals. In this study, barley grains were subjected to solid-state microbial fermentation using *Lactobacillus plantarum*, *Bacillus subtilis* and exogenous fibrolytic enzymes. This study aimed to assess the impact of FBEs on growth, feed utilization efficiency, immune modulation, antioxidant status and the expression of intestinal barrier and nutrient transporter-related genes. One-day-old broiler chicks (Ross 308, *n* = 400) comprised four representative groups with ten replicates (10 chicks/replicate) and were fed corn-soybean meal basal diets with inclusions of FBEs at 0, 5, 10 and 15% for 38 days. Solid-state fermentation of barley grains with fibrolytic enzymes increased protein content, lowered crude fiber and reduced sugars compared to non-fermented barley gains. In consequence, the group fed FBEs10% had the superior feed utilization efficiency and body weight gain (increased by 4.7%) with higher levels of nutrient metabolizability, pancreatic digestive enzyme activities and low digesta viscosity. Notably, the group fed FBEs10% showed an increased villi height and a decreased crypt depth with a remarkable hyperactivity of duodenal glands. In addition, higher inclusion levels of FBEs boosted serum immune-related parameters and intestinal and breast muscle antioxidants status. Intestinal nutrient transporters encoding genes (GLUT-1, CAAT-1, LAT1 and PepT-1) and intestinal barriers encoding genes (MUC-2, JAM-2, occludin, claudins-1 and β-defensin 1) were upregulated with higher dietary FBEs levels. In conclusion, feeding on FBEs10% positively enhanced broiler chickens’ performance, feed efficiency and antioxidant status, and boosted intestinal barrier nutrient transporters encoding genes.

## 1. Introduction

The need for feed ingredients is expected to increase with the growing global demand for poultry meat and eggs. In consequence, the supply of cereal grains as a conventional energy source in poultry feed will not meet the growing demand for the poultry industry [1]. Barley (*Hordeum vulgare* L.) is considered as one alternative that can substitute common cereal grains in broilers’ feed. The greater nutritional value of barley grains is attributed to their larger endosperms (80% of the cereal grain) that comprise starch granules [2]. On the contrary, endosperm cell walls are consisted mainly of insoluble non-starch polysaccharides (NSPs) with high levels of β-glucans [3]. This higher content of NSPs in barley grains can create a physical barrier by encapsulating the nutrients and ultimately result in their limited feeding in poultry [4]. From this point of view, the search for new processing strategies of barley grains still necessitates close attention in order to overcome their limitations and increase inclusion levels in poultry diets, especially during periods of inadequate energy feed resources. In this context, one strategy to alleviate the anti-nutritive impact of NSPs and improve barley utilization, as well as augment the birds’ endogenous enzymes, is by adding different exogenous enzymes at the time of feeding [5]. Moreover, exogenous carbohydrases can produce fermentable oligosaccharides that are necessary for the better survival of probiotics in the gut [6]. Nevertheless, the optimal proficiency of these enzymes is restricted when added in feed, owing to their inadequate dietary retention in chickens’ guts and their limited activity in crop, gizzard and proventriculus [7].

The other new prospective strategy for boosting the nutritional characteristics of barley grains is via fermentation technology. Fermentation is a dynamic process aiming to break down complex molecules in feed into small simpler ones using microorganisms, substrates and environmental conditions [8]. Furthermore, fermentation can enhance the nutritional properties of feed ingredients [9,10] reducing the toxic elements and various anti-nutritional factors [11]. Fermentation technology using probiotic bacteria can enhance the concentration of probiotics, enzymes, organic acids, bioactive peptides and metabolites and may alter some compounds into more valuable components in fermented ingredients [12]. Additionally, a solid-state fermentation process with the aid of favorable fungi and bacteria can digest plant materials, which act as substrates for NSPs-degrading microorganisms and change them to nutritive feed ingredients [9,13]. Furthermore, microbial fermentation can prospectively strengthen broiler productivity, improve carcass quality and boost beneficial gut microbiota and immune resistance [14].

Lately, the supplementation of exogenous enzymes, particularly in the occurrence of microbial inoculants, have been revealed to augment the nutritional characteristics of fermented barley and wheat [15]. The main advantage from this synergistic effect is to facilitate the solubilization of plant cell wall into simple sugars that can be utilized as fermentable substrates by lactic acid bacteria and consequently improves its nutritional quality.

To the best of our knowledge, the synergistic beneficial impact combining microbial fermentation and the supplementation of exogenous enzymes on the barley’s nutritional value lacks sufficient investigation to this day. Hence, the main goal of this study was to elucidate the favorable effects, which have emerged from microbial fermentation, with the addition of exogenous enzymes to evaluate the effects of barley inclusions on growth performance, nutrients utilization efficiency, immune response, the redox status of intestine and breast muscles and the molecular aspects controlling intestinal barrier function and nutrient transporters of broiler chickens.

## 2. Materials and Methods

### 2.1. Ethical Approval

The management steps, the experimental conditions regarding the housing and care of birds and all related experimental procedures were conducted according to the Ethics Committee “Institutional Animal Care and Use Committee (ZU-IACUC/2/F/321/2022)” of the of Veterinary Medicine Faculty, Zagazig University.

### 2.2. Two-Stage Solid Fermentation of Barley with Exogenous Enzymatic Treatment

Barley fermentation was carried out using *Lactobacillus plantarum* (*L. plantarum* *FPS 2520*) and *Bacillus subtilis* *var. natto N21 (BS)*. For microbial stimulation, de Man, Rogosa and Sharpe (MRS) medium was employed for *L. plantarum* incubation. *B. subtilis* was incubated in tryptone soya broth at 37 °C in an Erlenmeyer flask (150 rpm). To activate *Aspergillus niger* (*A. niger*), Sabouraud dextrose agar (Oxoid Ltd., Basingstoke, UK) was utilized and incubated for 7 days at 24 °C. Fermentation was initiated by soaking barley in distilled water to attain 30% moisture content. Afterward, *B. subtilis* and *A. niger* (ATCC 9142) were added at concentrations of 10^6^ CFU/g and 10^6^ spore/g of feed, respectively, and with 10% water for 2 days of aerobic fermentation at 37 °C in the first phase. Then, *L. plantarum* was added (10^6^ CFU/g feed) for 5 days of anaerobic fermentation at 25 to 35 °C in the second phase. Exogenous enzymes, including beta-glucanase (200,000 IU/kg), beta-xylanase (100,000 IU/kg), pectinase (4,000,000 IU/kg), cellulase (40,000 IU/kg) and phytase (300,000 IU/kg) were commercially obtained (ALLAZYME-X, Huvepharma, Suite 230, Inc. 525 Westpark Drive, Peachtree City, GA, USA) and added at the initial step of fermentation (0.2 g/kg barley). Finally, fermented barley was dried to a moisture content of 9.10% using an oven at 50–60 °C for 72 h and then mixed with the feed ingredients.

### 2.3. Nutritive Value Evaluation of Fermented Barley

#### 2.3.1. Chemical Analysis and Microbial Assessment

Crude protein (CP) of feed was estimated by analyzing total nitrogen using the Kjeldahl method according to AOAC [16] and the CP content of feed was estimated by multiplying the total N content by 6.25. Crude fiber, neutral detergent fiber (NDF) and acid detergent fiber (ADF) were verified using a FIBERTHERM (C. Gerhardt GmbH & Co. KG, Cäsariusstraße, Germany) following the procedure of Van Soest et al. [17]. The ether extract (EE) was determined by calculating the extracted weight loss of dry matter (DM) by solvent diethyl ether via the Soxhlet extraction apparatus according to AOAC [16]. The calculation of hemicellulose was performed by subtracting ADF from NDF, whereas the difference between ADF and acid detergent lignin accounted for cellulose. A digital pH meter was used for measuring the pH values (PSH-3C, INESA Instrument, Shanghai, China).

For assessing total lactic acid bacterial count, one gram of each fermented and unfermented barley grain samples were separately mixed with sterile water (9 mL). In the next step, the previously prepared samples were diluted with buffered peptone water (10-fold).

For assessing *Lactobacilli* count, 100 µL of supernatant was added to De Man and Rogosa Sharpe agar (MRS, CM1153, Oxoid) and incubated at 37 °C for 48 h and 13% CO_2_,and Tryptone Soya Broth medium was used for assessing *Bacillus* spp. count at 37 °C.

#### 2.3.2. Analysis of Reduced Sugar

Reduced sugar was examined according to Miller [18]. In brief, a glucose standard solution (at a concentration of 10–100 µg/mL), 1 mL of 0.05 M acetate buffer at pH 4.8 and 1 g sample of fermented and unfermented barley were thoroughly blended. Afterward, dinitrosalicylic acid reagent (3 mL) in boiled bath water was added for 5 min at room temperature. The prepared mixture was cooled, and the absorbance was estimated with a UV–visible spectrophotometer at 540 nm.

#### 2.3.3. Phosphorus Analysis

Total phosphorus analysis was performed according to Kirkpatrick and Bishop [19] with a spectrophotometer. The ash of dried samples was dissolved in acids (perchloric and nitric) at standard method concentrations. After that, sample filtrates were used to measure the color absorption at 400 nm. Additionally, phytate phosphorus was measured using the Wade reagent/colorimetric method as defined by Gao et al. [20]. Briefly, phytate phosphorus was extracted in acid and reacted with Wade reagent before measuring the color reaction absorbance at 500 nm with a spectrophotometer (Spectronic Helios Gamma UV–Vis Spectrophotometer; Thermo Scientific, Waltham, MA, USA). The available phosphorus was calculated by subtracting the phytate phosphorus from the total phosphorus.

#### 2.3.4. Analysis of Phytase Enzyme Activity

The phytase activity was assessed by measuring the inorganic phosphorus released from sodium phytate, consistent with the standard steps of phytase analysis in animal feedstuffs (ISO/DIS 30024) standard [21]. The extraction of barley samples was performed using 0.2 M citrate buffer at room temperature for 30 min at pH 5.3. Samples were then filtrated, and the oily layer was eliminated. Pure filtrate was used to evaluate the phytase activity. The phytase activity unit was expressed as the amount of phytase required for 1 μg of inorganic phosphorus liberation in 1 min at pH 5.3 and 37 °C (FTU/kg).

### 2.4. Animals and Experimental Design

Four hundred one-day-old male broiler chicks (Ross 308) were weighed and allocated to four dietary treatments, each containing ten replicates with ten chicks/pen. The dietary groups comprised a control (on corn–soybean-based diet) and three other groups in which fermented barley with enzymatic treatment (FBEs) was included at levels of 5, 10 and 15%. The experimental diets were administered in a three-phase feeding program and included a starter (day 1–10), grower (day 11–20) and finisher phase (day 21–38). The Aviagen Ross 308 management catalog Aviagen [22] guidelines were integrated to control the lighting, temperature and relative humidity in the experimental environment. Dry feed and water were provided ad libitum to each pen. Experimental diets were formulated as per the Ross broiler nutrition specifications (Table 1, Table 2 and Table 3). The proximate analysis of the feedstuff ingredients was performed following the standard approach of the AOAC [16]. The analyzed CP contents in soybean meal, corn and corn gluten were 47.9, 9.8 and 59.6, respectively. Meanwhile, the crude fiber contents in soybean meal, corn and corn gluten were 3.70, 2.30 and 1.8, respectively. Birds were fed crumble diets in the starter (1.5–2.4 mm) and pelleted diets in grower and finisher phases (3.5 mm) using a steam pelleting machine (Koppers Junior C40, Inc., Pittsburgh, PA, USA). The conditioning time was approximately 10 s at 75 °C and under a pressure of 1.5 kg/cm^2^ form as defined by the nutrition specifications in the Aviagen Ross broiler handbook, Aviagen [22].

### 2.5. Growth Performance and Nutrient Metabolizability Trial Monitoring

Average body weight (BW) of representative birds and feed intake were assessed throughout the starter, grower and finisher periods. Body weight gain (BWG) and feed conversion ratio (FCR) were assessed for each feeding phase and for the entire rearing period over 38 days.

The estimation of metabolizability of nutrients was performed according to Souza et al. and Zhang et al. [24,25]: titanium oxide (TiO_2_) was used as an indigestible marker, where three grams was mixed with each kg of experimental diet (finisher). The total excreta were collected from chickens for seven days with exclusion of any contaminants and then dried for 72 h at 65 °C and kept at −20 °C. The content of TiO_2_ in diets and excreta was determined after acid digestion. The total tract metabolizability calculations were performed according to AOAC [16] for DM, CF, excreta metabolizability of CP and phosphorus availability. Nutrient metabolizability% = 100 − [100 × (dietary indicator quantity (g)/fecal indicator quantity (g) × nutrient quantity in excreta (g)/nutrient quantity in feed (g)].

### 2.6. Sampling

At 38 days of age, the experimental birds (*n* = 5/group) were randomly picked and euthanized via cervical dislocation to collect blood samples and separate the sera via centrifugation for 15 min at 3000 rpm. The separated serum samples were stored at −20 °C until further analysis of immune and lipid-related parameters. Afterward, the eviscerations of the euthanized birds were performed for collection of various tissue samples. Homogenization of collected jejunal and breast muscle tissues were performed in ice-cold phosphate buffer saline (PFS; pH 7.4) and 20 mL of glycerol, followed by centrifugation at 3000 rpm for 15 min. Eppendorf tubes were used to collect the supernatants, which were used for the analysis of antioxidant capacity. For the digestive enzymes’ examination, pancreatic tissues were immediately collected after euthanasia (*n* = 5), minced in ice-cold PFS and centrifuged at 3000 rpm for 15 min, and the supernatants were used for analysis. Duodenal tissues (*n* = 5/replicate) were flushed with PFS and kept at −80 °C in Eppendorf cap-lock tubes to extract RNA. Samples of intestinal contents were immediately collected to estimate digesta viscosity and pH.

### 2.7. Digesta Viscosity and Duodenal pH

The jejunal digesta viscosity was measured as described by Perera et al. [26]. Briefly, collected digesta samples were centrifuged at 3000 rpm at 20 °C for 10 min. Viscosity was assessed in the supernatant (0.5 mL) with a viscometer (Brookfield digital viscometer, Stoughton, MA, USA) supplied with a CP-40 spindle with 5 to 500/s shear rates. The pH of the duodenal content was evaluated with a digital pH meter (PSH-3C, Shanghai, China).

### 2.8. Digestive Enzymes Estimation

The supernatants obtained from pancreatic samples were employed to measure the digestive enzyme activities using commercial diagnostic kits (Nanjing Jiancheng Bioengineering Institute, Nanjing, China) as per the manufacturer’s instructions.

### 2.9. Serum Biochemical Investigations

To estimate the lysozyme and nitric oxide (NO) activities, commercial kits (Jiancheng Biotechnology Institute, Nanjing, China) were used. Serum immunoglobulin IgG and IgM measurements were performed using enzyme-linked immunosorbent assay kits (Enzyme-linked Biotechnology Co., Ltd., Shanghai, China) following the provided guidelines. Creatinine, uric acid, cholesterol, triglycerides, low-density lipoprotein (LDL), high-density lipoprotein (HDL), alanine transaminase (ALT) and aspartate transaminase (AST) values were measured using standard commercial kits (Span Diagnostic Ltd., Sachin, India).

### 2.10. Measurement of Redox State in Jejunum and Breast Muscle

Serum superoxide dismutase (SOD, 19160), catalase (CAT, 219265) and glutathione peroxidase (GSH-Px, CS0260) activities were estimated using Sigma assay kits following the company’s guidance. Lipid peroxidation (MDA) was evaluated using the NO, MAK085 assay kit from Sigma-Aldrich. Muscle hydrogen peroxide (H_2_O_2_) amounts were determined as per Fernández-Puente et al. [27], and the obtained values were estimated as μmoL/g of tissue. To assess the (reactive oxygen species) ROS content in meat, the oxidation technique of Pranczk et al. [28] was utilized. The total antioxidant capacity (T-AOC) was established using a commercial kit (Sigma-Aldrich, MAK187, St. Louis, MO, USA).

### 2.11. Quantification of Intestinal Barrier Function and Nutrient Transporter-Associated Genes via RT-qPCR

Intestinal tissues were used for assessing the mRNA expression levels of genes’ encoding barrier functions (junctional adhesion molecule-2 (JAM-2), mucin-2 (MUC-2), occludin, claudins-1 (CLDN-1) and β-defensin 1) and nutrient transporters’ encoding genes (glucose transporter-1 (GLUT-1), cationic amino acid transporter-1 (CAT-1), peptide transporter-1 (PepT1)). RNA extraction was carried out using QIAamp RNeasy Mini kit (Qiagen, Hilden, Germany) as described by the manufacturers’ directions. The RNA quantification was assessed at 260 nm and the clarity of extracted RNA was spectrophotometrically determined by computing the absorbance wavelength ratio at 260–280 nm. After that, one-step RT-qPCR assay was completed using a QuantiTect SYBR Green RT-PCR Kit (Qiagen, Hilden, Germany) on the Strata-gene MX3005P real-time PCR recognition system. The precision of all PCR amplifications was established via the exploration of melting curve. The transcript expression level was standardized to the expression of those corresponding to TATA-binding protein (TBP), with glyceraldehyde 3-phosphate dehydrogenase (GAPDH) as endogenous controls. The sequences of gene-targeted primer employed in RT-qPCR assay are stated in Table 4. The relative mRNA expression results of explored genes were assessed via the 2^−∆∆Ct^ according to the method of Livak and Schmittgen [29].

### 2.12. Histopathological Examination of Intestine

Five birds per treatment were euthanized and duodenum samples were immediately collected (segments of approximately 3 cm from the mid-section of the duodenum). The specimens were immersed in 10% neutral buffer formalin, then processed using the usual histopathological technique to be immersed in paraffin wax [30]. Specimens were sectioned at 5–7 µm in thickness, stained by H&E stains, and then slides of tissue micro-sections were examined using a light microscope. Crypt depth was determined from the base upward to the region located between the crypt and villus [31]. Villus height was measured from the top of the villus to the top of lamina propria [32]. These images were analyzed utilizing Image J (version 1.50i) software.

### 2.13. Statistical Analysis

In the current study, homogeneity was verified with Levene’s test, and normality was tested using the Shapiro–Wilk test with the model Y_i_k = μ + L_i_ + e_i_k, where Y_i_k is the estimation, μ is the total mean, L_i_ is the impact of the experimental treatments and eik is the random error. All statistics-related data were explored using the GLM procedure of SPSS version 22. Deviations in the data were defined as standard error of the mean (SEM), and significance was denoted at *p* ≤ 0.05. Tukey’s test was employed to evaluate the significant variations in the mean values. All graphs were created using GraphPad Prism software (version 8).

## 3. Results

### 3.1. Chemical Analysis of Unfermented Barley Grains and Fermented and Enzymatically Treated Barley Grains

As presented in Table 5, the crude protein and reduced sugar contents were significantly increased after the fermentation of barley grains; nevertheless, crude fiber, lignin, cellulose, NDF and ADF (*p* < 0.003) reduced after fermentation. Notably, phytase activity (*p* < 0.001) and non-phytate phosphorus (*p* < 0.007) in FBEs decreased when compared with unfermented barley grains. Regarding the microbial population, the total lactic acid bacteria and *Bacillus* spp. count significantly increased in FBEs.

### 3.2. Growth Performance

The impact of the dietary inclusion of various levels of FBEs on the growth performance of broiler chicks throughout the experimental period is depicted in Table 6. At the end of the stater period, BWG and FCR improved (*p* < 0.004) in the group fed with FBEs at the level of 10%, with no significant differences found among other experimental groups. The dietary inclusion of FBEs did not influence the feed intake of broilers during days 0 to 10. Moreover, the BWG and FCR in the grower period enhanced (*p* < 0.040) in the groups fed with 10 and 15% FBEs in contrast to the control group. The feed intake in the grower period decreased (*p* < 0.040) in the group fed with 10% FBEs unlike the control group. At the end of the finisher period, a higher BWG and superior FCR were found in the group fed with 10% FBEs followed by the groups fed with 5 and 15% FBEs in comparison with the control group. Regarding the overall growing period, the dietary inclusion of FBEs boosted the overall growth performance parameters of broiler chickens when compared to the control group. Moreover, broilers fed with 10% FBEs displayed the most prominent improvement (*p* < 0.030) in BWG and FCR (*p* < 0.001).

### 3.3. Estimating Nutrient Metabolizability and Activities of Pancreatic Digestive Enzymes

The dietary inclusion effect of FBEs on nutrient metabolizability percent (%) and digestive enzyme activities are presented in Table 7. A significant increase in DM (*p* < 0.001), CP (*p* < 0.020) and crude fiber (*p* < 0.030) metabolizability were detected, following the feeding of FBEs, especially at the 10% inclusion level, unlike control group. Phosphorus availability in ileum (*p* < 0.040) was considerably elevated in all groups fed with different levels of FBEs. Moreover, the duodenal pH (*p* < 0.030) decreased simultaneously with increasing FBE levels in diets. Jejunal digesta viscosity (*p* < 0.060) showed differences among different experimental groups. The inclusion of different levels of FBEs increased the activities of pancreatic lipase and amylase (*p* < 0.001). Broilers fed with FBEs at the level of 10 and 15% (*p* < 0.010) showed an increase in intestinal maltase and sucrase activities. Increasing the inclusion level of FBEs enhanced trypsin (*p* < 0.030) and chymotrypsin (*p* < 0.01) activities when compared with control group.

### 3.4. Estimating Immune and Biochemical-Related Parameters

The immune response and biochemical parameters of broilers supplemented with various levels of FBEs are illustrated in Table 8. With increasing levels of FBEs in broiler diets, IgG (*p* < 0.090) and lysozyme (*p* < 0.001) increased but inversely when the nitric oxide levels were reduced. Higher levels of IgM were noticed in the group supplemented with 15% FBEs. Compared to the control, various substitution levels of FBEs had no significant effect on uric acid, creatinine, AST and ALT. The noticeable reduction in the levels of cholesterol, triglycerides and LDL-c and the elevation in levels of HDL-c were observed in the group fed with FBEs at the level of 15%.

### 3.5. Antioxidant Functions of FBEs on Intestinal Tissues and Breast Muscle

The biomarkers of the antioxidant and oxidative stability of intestinal tissues and breast muscles are presented in Table 9. The selected antioxidant enzymes in intestinal tissues (GSH-PX, SOD and CAT) showed an increased activity in response to higher inclusion levels of FBEs. Moreover, their activities were significantly elevated in a dose-dependent pattern with increasing levels of dietary FBE inclusion in breast muscle. Regarding T-AOC, their levels were elevated (*p* < 0.001) in intestinal tissues and breast muscle in groups fed with 10 and 15% FBEs when compared with control group. The intestinal and breast muscle contents from ROS and H_2_O_2_ lowered with the rise in the inclusion levels of FBEs. The lowest level of lipid peroxidation biomarker (MDA) was obviously detected in intestinal tissues and breast muscle in the group fed with 15% of FBEs.

### 3.6. Intestinal Nutrient Transporter-Related Genes Expression

**Figure 1:**
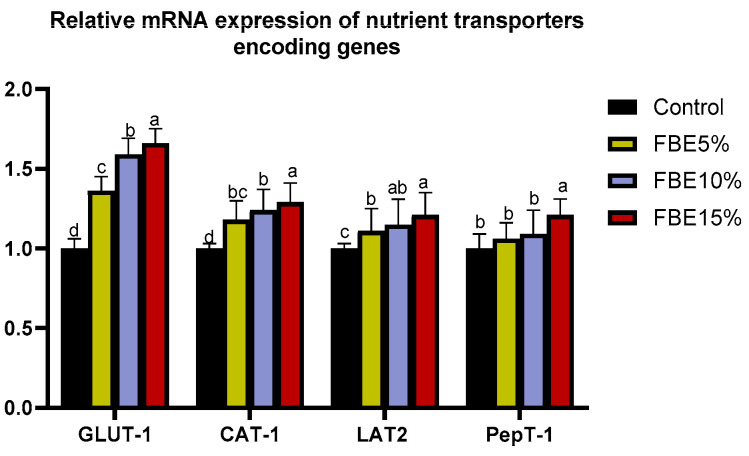
Effects of different levels of microbially fermented barley treated with exogenous enzymes (FBEs) on the expression of duodenal genes (GLUT1, Cationic amino acid transporter-1 (CAT-1), L-type amino acid transporter-1 (LAT2) and Peptide transporter-1 (PEPT1)). Control: birds fed on basal diet; FBEs5% (birds fed dietary 5% microbially fermented barley treated with exogenous enzymes), FBEs10% (birds fed dietary 10% microbially fermented barley treated with exogenous enzymes), FBEs15% (birds fed dietary 15% microbially fermented barley treated with exogenous enzymes). ^a–d^ Means within the equivalent column with dissimilar superscripts are significantly diverse (*p* < 0.050).

The higher inclusion levels of FBEs upregulated (*p* < 0.050) the mRNA expression of GLUT1 and GLUT2 (Figure 1). Regarding the proteins that related to transporters genes, groups fed with 10 and 15% of FBEs had an upregulated level of CAT-1. Additionally, the most remarkable mRNA expression of PEPT-1 was observed in the group fed with FBEs at the level of 15%.

### 3.7. Intestinal Tight Junction-Related Genes Expression

**Figure 2:**
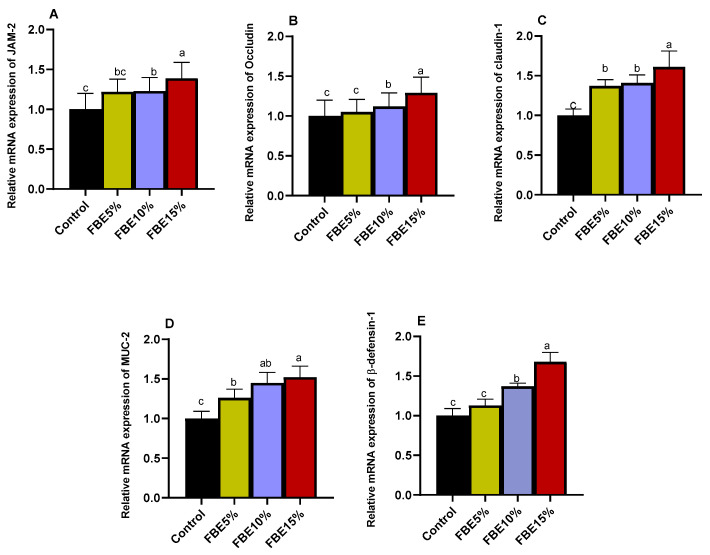
Effects of different levels of fermented and enzymatically treated barley grains (FBEs) on the expression of duodenal tight junction genes JAM (junctional adhesion molecule-2, (**A**)), occluding (**B**), claudin-1 (**C**), MUC-2 ((**D**), mucin-2) and β-defensin-1 (**E**). Control: birds fed on basal diet; FBEs5% (birds fed dietary 5% microbially fermented barley treated with exogenous enzymes), FBEs10% (birds fed dietary 10% microbially fermented barley treated with exogenous enzymes), FBEs15% (birds fed dietary 15% microbially fermented barley treated with exogenous enzymes). ^a–c^ Means within the equivalent column carrying dissimilar superscripts are significantly diverse (*p* < 0.050).

Real-time qPCR analysis results for JAM, occludin, claudin-1, MUC-2 and β-defeinesin-1 are shown in Figure 2. Compared with the control group, all FBE-fed groups exhibited an elevation (*p* < 0.050) in the levels of the mRNA expression of tight junction-associated genes, with the ultimate significant level obtained for the 15% FBE group (increased by 1.39-, 1.29-, 1.61-, 1.52- and 1.68-fold compared to control group).

### 3.8. Evaluating Intestinal Histomorphology in Response to Feeding on FBEs

**Figure 3:**
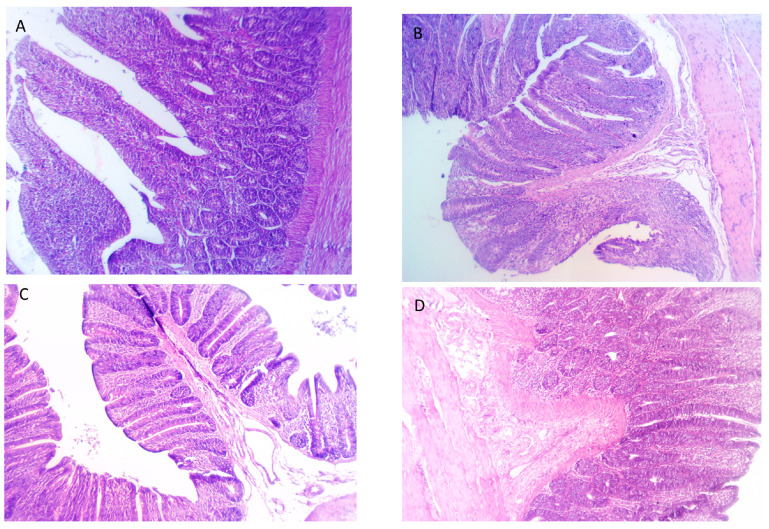
Effects of feeding different levels of fermented and enzymatically treated barley grains (FBEs) on duodenal histomorphological architectures. (**A**) Control: Intestine showed normal intestinal villi, lamina propria, submucosa, intestinal glands and muscular layer; (**B**) FBEs5%-fed birds: Sections from intestine showed apparently normal intestinal villi, submucosa and muscular layer; (**C**) FBEs10%-fed birds: showed an increased activity of columnar lining epithelium of villi, normal lamina propria, submucosa, muscular layer and serosa; (**D**) FBEs15%-fed birds: intestine showed apparent normal intestinal layers with numerous and hyperactive intestinal glands. H&E 10×.

The impact of feeding broiler chickens on FBEs (*p* < 0.050) influenced the villi length, crypt depth and their ratio in the duodenum compared to control (Table 10). The supplementation of the broiler’s diets with FBEs10% heightened the villi length and lessened crypt depth in the duodenum (*p* < 0.050), up to 6.78% and 28.18%, respectively. A significant increase in the duodenal villi height: crypt depth ratio was detected with an increasing dietary inclusion of FBEs up to 10%. As described in Figure 3A,B, duodenum showed normal villi, glands, lamina propria, submucosa and the muscular layer. Meanwhile, birds fed with FBEs10% showed an increased activity of the columnar lining epithelium of villi, normal lamina propria, submucosa, muscular layer and serosa (Figure 3C). For the FBEs15%-fed birds, as described in Figure 3D, duodenum layers were apparently normal layers with numerous and hyperactive glands.

## 4. Discussion

The most common conventional cereals such as corn and wheat are predicted to not meet the future demand of the animal feed sector in the modern fast-growing poultry industry. This overdemand not only exerts pressure on the feed market’s raw materials, but also encourages the evaluation of alternative feed materials that can be included in poultry diets [33]. The dietary inclusion of barley grains is low in poultry diets due to their high content of anti-nutritional factors such as NSPs and phytic acid [34]. Additionally, the degradation of NSPs in barley-based diets by exogenous fibrolytic enzymes can overcome the adverse consequences of NSPs on the birds’ utilization of nutrients and performance [35]. These limitations to using barley as an energy source in poultry diets require searching for novel processing strategies to increase its dietary inclusion levels. Applying solid-state fermentation technology can improve the inclusion rate of raw plant materials with low nutritional characteristics such as barley grains in animal feed [36].

Microbial fermentation is an effective technology for improving barley’s nutrient metabolizability and nutrient assimilation in poultry and other monogastric animals. Using the synergistic impact of probiotics and enzymes to predigest feed is better than using single fermentation or enzymatic hydrolysis alone by enhancing macromolecule degradation and microbial fermentation efficiency [37]. The crude protein content in fermented barley increased by 10.88% compared with non-fermented barley, which may be due to the fermenting microorganisms using the carbonaceous substrates in the grains as energy sources to produce microbial protein. Moreover, fermenting microorganisms with additional exogenous enzymes can depolymerize the fibrous content of plant cell walls, use it to produce nitrogenous compounds and alter protein solubility, improving plant proteins’ degradability [38,39]. Correspondingly, Liu et al. [40] stated that the solid-state fermentation of different barley grain forms (germinated, soaked and unsoaked) increased protein and amino acid contents, especially valine, alanine, phenylalanine, aspartic acid and glutamic acid. A similar study proved that lactic acid bacteria fermentation produces proteases that hydrolyze proteins and elevate the free amino acids content [41].

Barley grains are an essential feedstuff used in animal feeding characterized by its 11–13% hull and 4–6.0% total fiber contents and considerable NSP content, comprising mostly pentosans and β-glucans [42]. Carbohydrates in barley are not as easily digested as that in corn as the fiber in corn contains lower levels of NSP, such as β-glucans, lignin and cellulose, compared to barley grains.

The crude fiber and NSP constituents of barley can be degraded via ruminal fermentation, while monogastric animals do not have adequate enzymatic and microbial activities to appropriately degrade them, causing the formation of viscous digesta in the digestive tract, which impairs the nutrients’ absorption [43]. Solid-state fermentation has been reported as an effective approach for improving the nutritive value of cereals by reducing the cellulose content and improving the acid-soluble protein content [44,45]. Our study showed that the FBEs nutritive content was altered after solid-state fermentation using multi-exogenous enzymes that mainly acted on the fiber substrate (beta-xylanase- beta-glucanase, pectinase, xylanase and cellulase). These fibrolytic enzymes hydrolyze the polysaccharides in crude fiber into smaller carbohydrate units that act as a carbon source for the metabolic processes in energy production. Likewise, supplementation with exogenous fibrolytic enzymes during fermentation decreased the crude fiber content in FBEs, which accounted for the reduced biodegradation of lignin, cellulose, hemicellulose, NDF and ADF and increased reduced sugar content. Moreover, Xiao et al. [46] described that the degradation and hydrolysis of cellulose and hemicellulose and the loosening of lignin bonds increased the soluble polysaccharides and decreased the insoluble fibrous content due to the solid-state fermentation process with added exogenous enzymes. In agreement, Shi et al. [36] indicated that NDF, hemicellulose and phytic acid were reduced after the microbial fermentation of corn–soybean meal, which could be attributed to enzyme secretion by microorganisms, such as those degrading NSPs and phytase. These modifications may be due to the fermentation products such as organic acids, various endogenous enzymes in the kernels, or bacterial enzyme production during the fermentation process.

Additionally, the majority of the phosphorous in barley remain in the form of phytate phosphorus that have low availability in monogastric animals [47]. In the current study, phytase activity increased after the fermentation of barley grains, which resulted in higher non-phytate phosphorus amount compared to unfermented barley grains. Yasar and Tosun [48] described that the supplementation of exogenous enzymes, including phytase, during the solid-state fermentation process to barley grains successfully increased phytase enzyme content and their activity consequently increased non-phytate phosphorus quantity, which is in agreement with our finding. A reduced phytic acid amount in fermented feed may be resulted from an increased microbial phytase production during fermentation [49]. Among a variety of factors, the pH of fermented feed is an essential parameter to assess its quality, since lowering the pH may promote nutrient digestion in the intestine and prevent pathogenic microorganisms’ proliferation in feed [50]. Our results showed that the pH of FBEs decreased from 6.25 to 4.25, which indicated good-quality fermented barley. This decrease in pH resulted from a higher lactic acid production as the pH may have declined only when the cereals were fermented because they have a minimal buffering capability than compound feed [51]. Also, lowering the pH was due to the higher proliferation of lactic acid-producing bacteria in fermented barley, which is consistent with Shi et al.’s findings of an increased count of total lactic acid bacteria and *B. subtilis* count after the fermentation process [36].

Replacing corn with raw barley grains reduced the BWG of broiler chicks during the starter period [6]. However, in the current study, substituting corn with fermented barley up to FBEs10% in diets for broiler chicks resulted in a noticeable improvement in the growth performance, which can be explained by the synergistic positive impact of adding enzymes and fermentation barley grains that enhanced their nutritional value and increased the availability of nutrients for the birds. Also, Li et al. [52] described that broilers fed with numerous ratios of feed fermented with *Lactobacillus* spp. and *B. subtilis* at the level of 10% increased the average daily gain of broilers and their feed efficiency. Skrede et al. [53] stated that the weight gain of broiler chickens fed with fermented barley and fermented wheat without the addition of commercial enzymes was greatly improved compared to those fed a control diet. Similarly, feeding on fermented feed at the level of 10% boosted the FCR of broilers and tended to increase the body gain compared to those fed with 20% and 0% for fermented feed [54].

In addition, the inclusion of a barley-based diet as well as a cocktail of NSPs-degrading enzymes as a substitute to a corn–soy-based diet effectively improved BWG and feed utilization in broiler chickens [55]. The additional advantages of feeding on FBEs included an enhanced nutrient metabolizability (DM, CP and CF) and phosphorus bioavailability by augmenting the substrate availability, which accelerated the activity of digestive enzymes in the broiler chickens, which consequently agrees with the findings of Shi et al. [36]. Accordingly, dietary FBE inclusion enhanced the building of carbohydrates and protein by raising the nutrient transport proficiency in intestinal epithelial cells, which follows the findings of Horvatovic et al. [56]. Compared with a corn-based diet, adding carbohydrases to barley during fermentation enhanced the activities of starch digestive enzymes (amylase, maltase and sucrase), thus improving the metabolizability of barley-based diets for broilers, which agrees with the findings of Perera et al. [26,57]. As such, the fermentation process can degrade macromolecular factors and anti-nutritional elements in raw feed stuff via microorganisms, thereby facilitating the feed’s metabolizability and absorption, with an improvement in the subsequent growth performance of the broilers [58]. Furthermore, applying feed fermentation technology to broiler feed not only decreases anti-nutritional substances, but also augments the concentrations of beneficial probiotic bacteria, enzymes and fermentation metabolites [59,60]. Meanwhile, the lowered feed and duodenal pH found in the current study could be related to the enhanced proliferation of beneficial probiotic bacteria such as *Lactobacillus* and *Bacillus* spp. in fermented barley that might encourage the degradation of compound carbohydrates, especially cellulose and hemicelluloses, into organic acids [61,62].

Lactic acid produced via fermentation can decrease the pH of feed and the digestive tract, which provides a good environment for phosphorus absorption. This may be because the beneficial microorganisms in the fermentation broth can produce some metabolites, such as lactic acid, bacteriocin, antibacterial substances, higher alcohols which can reduce the pH of the digestive tract, inhibit or kill harmful bacteria (such as *Escherichia coli*) and improve the digestion and absorption capacity of the intestines [63]. The enhanced broiler growth performance in the current study could be associated with the improved nutritive values of FBEs. Moreover, the starch content in corn ranges from 65 to 70% and is easily digested, while the starch content in barley is approximately 60% and proven to not be easily digested in monogastric animals and poultry [64]. Therefore, barley grain inclusion in broilers’ diets, especially in the starter phase, is limited as it causes an increase in digesta viscosity and lowered nutrient absorption, which cause growth performance retardation in broiler chickens [65]. Therefore, the fermentation technology with exogenous additions showed its maximum efficiency in broiler performance when including up to 10% of FBEs. Even though feeding on FBEs decreases the adverse effects of NSPs content in barley grains, a higher inclusion level in broiler diets, especially in the starter stage, may exceed the birds’ capability to digest it efficiently, with a consequent retarding effect on growth performance [34]. Moreover, this explanation is supported by the nutrient metabolizability analyzed data, which showed higher values of up to 10% FBE inclusion.

Accordingly, the duodenum histomorphological images revealed an increased villus height and lowered crypt depth after feeding on FBEs, especially at the 10% level. Similarly, birds fed with solid-state fermented feed with *Lactobacillus casei* had a greater villus height and lower crypt depth in the duodenum than chickens fed with the control diet, as well as a greater villus height/crypt depth ratio in a study conducted by Peng et al. [66]. There is a strong correlation between enhanced nutrient digestion and improved intestine histomorphology as the function of intestinal villi improves with an increasing villus height/crypt depth ratio [67]. Our study revealed that the inclusion of 10% FBEs improved the villus height and villus height/crypt depth ratio, which follows the findings of Peng et al. [66,68]. In addition, El-Sanhoury and Ahmed [69] reported increased villus height and numbers after supplementation with exogenous enzymes containing cellulase and xylanase in broiler chickens. An explanation for the improved intestinal villus height and villus height/crypt depth ratio is that cereals containing high levels of NSPs increase the size of the gastrointestinal tract and change the intestinal morphology [70].

Other possible explanations for the enhanced broiler performance are that fermented barley-based diets treated with exogenous NSPs-degrading enzymes decrease the digesta viscosity, prompt the degradation of the cell wall, release the encapsulated nutrients, and induce gut microbiota modification via prebiotic oligosaccharides [71,72,73]. The viscous contents inside the intestines of birds fed with barley-based diets unlike birds fed with corn-based diets were noted due to their higher β-glucans content [74]. Higher levels of dietary NSPs can bind with a large amount of water, which increases the fluid viscosity and consequently hinders the digestion of nutrients, reducing their utilization [75]. An excess of insoluble fiber shortens the residence time of chyme in the intestines, and an excess of soluble fiber adheres to the surface of chyme to form a nutritional barrier, which is not conducive to the digestion of nutrients [76]. Herein, the viscosity of the intestinal digesta of broilers fed with FBEs up to 10% did not differ from those fed a corn-based diet, which is supported by the findings of Bedford [71].

Nutrient absorption in the small intestines is mainly intermediated by transporter proteins expressed in enterocytes. The upregulation of these transporters boosts nutrient transportation efficiency and enhances the influx of digested nutrients into the enterocytes and, later, to all body parts [77]. Herein, feeding broiler chickens with FBEs upregulated the expressions of glucose (GLUT1) and amino acid (CAT-1, LAT2 and PEPT1) transporter genes. These results agree with those of Al-Khalaifah et al. [78], who reported that feeding broiler chickens with enzymatically treated fermented dried brewer’s grains upregulated amino acids and all glucose transporter encoding gene expressions. Following our results, supplementing a reduced-energy feed with enzymes in broiler diets boosted micronutrient absorption via GLUT-2 and PEPT1 upregulation [79]. Additionally, using carbohydrate-rich feed can greatly upregulate the expression of glucose transmitters and consequently increase the absorption of glucose [80]. Moreover, xylanase enzyme supplementation in broilers’ diets was reported to upregulate the expressions of GLUT2 and PEPT1, which positively impacted nutrient absorption [81,82].

The intestinal barriers can protect the intestine from pathogen colonization and adhesion [83]. Fermentation products, including probiotics [84], have been demonstrated to upregulate the gut barrier-related genes in poultry, but their mechanisms of action need further investigation. Our results showed a significant improvement in the expression of intestinal JAM, occludin, claudin-1, MUC-2 and β-defensin-1 with increasing levels of FBEs, suggesting the positive impact of fermented feed on gut barrier functions. OCLN and zona OCLN can produce a gut extracellular barrier [85,86], which are the main tight junction proteins that provide protection against invasive intestinal pathogens [87]. MUC2 is an essential gene responsible for mucin secretion in the intestinal mucosa that control the attachment sites for host bacteria [85,88].

The main microbial fermentation metabolites include prebiotic-like compounds such as oligosaccharides that have been proven to have an immune-boosting function [89]. These probiotic and prebiotic effects of FBEs might have the potential to not only enhance the growth performance, but also modify the gastrointestinal ecosystem, metabolic activities and immune response. Serum immunoglobulins are the key indicators of an animal’s humoral immunity as they play crucial roles in defending against pathogenic microorganisms [90,91]. Herein, serum IgG and IgM concentrations increased in response to the increase in the level of fermented barley in the feed. Previous studies have proven that the elevation in immunoglobulin concentrations in the serum of broilers fed with fermented feed may be associated with the production of small-sized peptides and the increase in amino acid concentration during fermentation, which act as a substrate for immunoglobulin synthesis [92,93]. Another explanation might be that fermented feed inclusion increases the source of beneficial probiotic bacteria, such as *Lactobacillus*, which has been verified to stimulate the production of immunoglobulin in broilers [94]. Similar results were obtained by Xu et al. [95], who reported that dietary supplementation with fermented feed in broiler chickens increased serum immunoglobulin levels. Serum lysozyme plays an important role in the lysis of invading Gram-positive bacteria by hydrolyzing the b-1,4 glycosidic bonds of peptide glycans, which destroys the murein layer of the bacterial cell wall and reduces its mechanical strength, resulting in the destruction and lysis of the bacteria [96]. In our study, including fermented barley in the feed stimulated the lysozyme activity in broiler serum. Agreeing with our findings, Zhu et al. [97] proved that fermented feed supplementation increased serum lysozyme activity. Nitric oxide (NO) is a typical free radical and pro-oxidant produced via the oxidation of L-arginine by nitric oxide synthase in macrophage cells in response to inflammation. Nitric oxide can freely pass through the cell membrane and shows strong oxidation activity [98]. An excessive amount of NO production can cause tissue damage via reactive nitrogen and oxidative stress effects [99]. Herein, the NO levels decreased by increasing the inclusion levels of FBEs, which is consistent with the findings of Wu et al. [94].

Liver health status can be indirectly estimated by serum concentrations of AST and ALT, as higher levels are considered markers for liver damage [100]. Furthermore, kidney function can be anticipated by measuring up-normal variations in urea and creatinine serum levels. Our concurrent results show that dietary FBEs had no significant impact on serum AST, ALT, uric acid and creatinine levels, which were within the normal ranges, indicating healthy liver and kidney functions in the FBE groups. Moreover, the highest reductions in total cholesterol and triglyceride concentrations in the broiler serum were detected in the group fed with 15% FBEs, which was attributed to the hypocholesterolemic effects of probiotics resulting from microbial fermentation [101]. Probiotic strains may boost bile salt hydrolase activity, resulting in bile salt deconjugation, and can uptake cholesterol into their cells with a subsequent decrease in the cholesterol level in the surrounding environment [102].

Intestinal tissue and breast muscle concentrations of antioxidant enzymes and lipid peroxidation biomarkers such as MDA and T-AOC reflect the general antioxidant status and consequently the health of birds. Herein, increasing the antioxidant capacity of intestinal tissues and breast muscle and enhancing their capacity to scavenge free radicals demonstrated the antioxidant role of FBEs. A higher production of fermented metabolites with antioxidant potential in fermented feed has been proven to enhance its functional properties [103,104,105]. Furthermore, the higher antioxidant efficacy in fermented products could result from various active peptides generated from the protein hydrolysates in the substrates and the production of phenolic compounds that counteract free radicals [106,107]. MDA is an end-product of lipid peroxidation; hence, its level can be used to monitor the degree of lipid peroxidation [108]. The decline in the MDA concentration in our experiment was associated with a drop in the level of lipid peroxidation end-products. In accordance, fermentation products such as microbial glucosidases are more effective at scavenging free radicals [109]. Furthermore, dietary probiotics have been verified to promote antioxidant enzyme activities and reduce the harmful impacts of oxidative stress [110]. Antioxidant compounds such as phenolic and flavonoid complexes, T-SOD, catalase enzymes, GSH-Px activities and total antioxidant capacity were increased, and MDA generation was inhibited in hepatic tissues and serum in response to microbially fermented soybean meal with *Bacillus amyloliquefaciens* [111]. In broiler chickens’ diets, the addition of solid-state fermented Isaria cicadae enhanced the activities of GSH-Px, T-SOD and T-AOC due to the presence of polyphenols with strong free radical scavenging activity [112,113].

## 5. Conclusions

The beneficial impacts that emerged from the fermentation of barley grains with probiotics and exogenous enzymes not only improved their nutritive quality, but also promoted the birds’ performance due to the health-boosting benefits, augmenting feed utilization. The beneficial molecular findings of gene expression regulating the intestinal barrier and nutrient transports can support the favorable use of higher-dietary-inclusion levels of FBEs in the diets of broiler chickens. Herein, our outcomes described that the building up of strong antioxidant qualities and the immune system after feeding on FBEs can provide a new sense of hope to minimize the oxidative stress in poultry farms. Taken together, our study recommended that a dietary inclusion of 10% FBEs enhances birds’ performance and improves the feed efficiency of broiler chickens.

## Figures and Tables

**Table 1:** Levels of ingredients and nutrients in diets throughout the starter phase (as fed basis).

Ingredients	Control	FBEs5%	FBEs10%	FBEs15%
Yellow corn	58.80	55.30	50.30	45.80
Soybean meal, 48% CP	34.00	32.50	32.50	32.00
Corn gluten	1.20	1.20	1.20	1.20
FBEs ^1^	-	5.00	10.00	15.00
Soybean oil	1.80	1.80	1.80	1.80
Calcium carbonate	1.20	1.20	1.20	1.20
Calcium diphasic phosphate	1.50	1.50	1.50	1.50
Common salt	0.30	0.30	0.30	0.30
Premix ^2^	0.30	0.30	0.30	0.30
L-Lysine	0.35	0.35	0.35	0.35
DL-Methionine	0.25	0.25	0.25	0.25
Choline chloride	0.20	0.20	0.20	0.20
Anti-mycotoxin	0.10	0.10	0.10	0.10
Analyzed and calculated composition				
Metabolizable energy (kcal/kg) ^3^	3048	3038	3015	3000
Crude protein (%)	23.40	23.07	23.16	23.06
Ether extract%	4.28	4.22	4.12	4.03
Crude fiber (%)	2.63	2.67	2.70	2.75
Calcium (%)	1.04	1.04	1.03	1.03
Available phosphorous (%)	0.46	0.49	0.54	0.57
Lysine (%)	1.33	1.30	1.30	1.29
Methionine (%)	0.58	0.58	0.57	0.57

^1^ FBEs (microbially and enzymatically fermented barley), FBEs5% (dietary inclusion of 5% microbially and enzymatically fermented barley), FBEs10% (dietary inclusion of 10% microbially and enzymatically fermented barley), FBEs15% (dietary inclusion of 15% microbially and enzymatically fermented barley). ^2^ Vitamin premix supplied per kilogram of diet: vitamin A, 10,000 IU; vitamin D3, 2000 IU; vitamin E, 6500 IU; vitamin K3, 1 mg; vitamin B1, 2560 mg; vitamin B2, 5000 mg; vitamin B6, 1500 mg; B5, 8 mg; niacin, 20,000 mg; biotin, 0.25 mg; folic acid, 1000 mg; vitamin B12, 60 mg; Cu, 8 mg; Fe, 80 mg; Mn, 60 mg; Zn, 40 mg; Se, 0.15 mg. ^3^ Calculated based on Janssen’s equation [23].

**Table 2:** Levels of ingredients and nutrients in diets throughout the grower phase (as fed basis).

Ingredients	Control	FBEs5%	FBEs10%	FBEs15%
Yellow corn	63.30	59.30	55.20	50.80
Soybean meal, 48% CP	27.90	26.90	26.00	25.40
Corn gluten	2.00	2.00	2.00	2.00
FBEs ^1^	-	5.00	10.00	15.00
Soybean oil	2.50	2.50	2.50	2.50
Calcium carbonate	1.20	1.20	1.20	1.20
Calcium diphasic phosphate	1.50	1.50	1.50	1.50
Common salt	0.30	0.30	0.30	0.30
Premix ^2^	0.30	0.30	0.30	0.30
L-Lysine	0.45	0.45	0.45	0.45
DL-Methionine	0.25	0.25	0.25	0.25
Choline chloride	0.20	0.20	0.20	0.20
Anti-mycotoxin	0.10	0.10	0.10	0.10
Analyzed and calculated composition				
Metabolizable energy (kcal/kg) ^3^	3122	3128	3113	3105
Crude protein (%)	21.30	21.30	21.14	20.95
Ether extract%	5.01	5.01	4.93	4.85
Crude fiber (%)	2.52	2.56	2.60	2.63
Calcium (%)	1.02	1.02	1.02	1.01
Available phosphorous (%)	0.50	0.54	0.58	0.62
Lysine (%)	1.25	1.25	1.23	1.22
Methionine (%)	0.56	0.56	0.57	0.55

^1^ FBEs (microbially and enzymatically fermented barley), FBEs5% (dietary inclusion of 5% microbially and enzymatically fermented barley), FBEs10% (dietary inclusion of 10% microbially and enzymatically fermented barley), FBEs15% (dietary inclusion of 15% microbially and enzymatically fermented barley). ^2^ Vitamin premix supplied per kilogram of diet: vitamin D3, 2500 IU; vitamin A, 20,000 IU; vitamin E, 6500 IU; vitamin K3, 1.4 mg; vitamin B1, 2580 mg; vitamin B2, 3500 mg; vitamin B6, 1900 mg; B5, 11 mg; niacin, 20,000 mg; biotin, 0.45 mg; folic acid, 1000 mg; vitamin B12, 66 mg; Cu, 9 mg; Fe, 85 mg; Mn, 60 mg; Zn, 46 mg; Se, 0.15 mg. ^3^ Calculated based on Janssen’s equation [23].

**Table 3:** Levels of ingredients and nutrients in diets throughout the finisher stage (as fed basis).

Ingredients	Control	FBEs5%	FBEs10%	FBEs15%
Yellow corn	68.00	63.00	59.00	54.90
Soybean meal, 48% CP	21.90	21.90	21.00	20.00
Corn gluten	2.50	2.50	2.50	2.50
FBEs ^1^	-	5.00	10.00	15.00
Soybean oil	3.40	3.40	3.40	3.40
Calcium carbonate	1.20	1.20	1.20	1.20
Calcium diphasic phosphate	1.50	1.50	1.50	1.50
Common salt	0.30	0.30	0.30	0.30
Premix ^2^	0.30	0.30	0.30	0.300
L-Lysine	0.35	0.35	0.35	0.35
DL-Methionine	0.25	0.25	0.25	0.25
Choline chloride	0.20	0.20	0.20	0.20
Anti-mycotoxin	0.10	0.10	0.10	0.100
Analyzed and calculated composition				
Metabolizable energy (kcal/kg) ^3^	3214	3205	3209	3199
Crude protein (%)	19.47	19.56	19.32	19.15
Ether extract%	6.08	5.95	5.90	5.83
Crude fiber (%)	2.41	2.47	2.50	2.53
Calcium (%)	1.01	1.01	1.01	1.00
Available phosphorous (%)	0.44	0.47	0.50	0.52
Lysine (%)	1.05	1.05	1.05	1.03
Methionine (%)	0.54	0.54	0.54	0.50

^1^ FBEs (microbially and enzymatically fermented barley), FBEs5% (dietary inclusion of 5% microbially and enzymatically fermented barley), FBEs10% (dietary inclusion of 10% microbially and enzymatically fermented barley), FBEs15% (dietary inclusion of 15% microbially and enzymatically fermented barley). ^2^ Vitamin premix supplied per kilogram of diet: vitamin D3, 2500 IU; vitamin A, 20,000 IU; vitamin E, 6500 IU; vitamin K3, 1.4 mg; vitamin B1, 2580 mg; vitamin B2, 3500 mg; vitamin B6, 1900 mg; B5, 11 mg; niacin, 20,000 mg; biotin, 0.45 mg; folic acid, 1000 mg; vitamin B12, 66 mg; Cu, 9 mg; Fe, 85 mg; Mn, 60 mg; Zn, 46 mg; Se, 0.15 mg. ^3^ Calculated based on Janssen’s equation [23].

**Table 4:** Primer sequences of targeted genes used for RT-qPCR.

Encoding Gene	Primer Sequence (5′–3′)	Accession No.
Barrier functions		
β-Defensin-1	F: AAACCATTGTCAGCCCTGTG R: TTCCTAGAGCCTGGGAGGAT	NM_204993.1
MUC-2	F: AAACAACGGCCATGTTTCAT R: GTGTGACACTGGTGTGCTGA	NM_001318434
CLDN*-1*	F: GGTGAAGAAGATGCGGATGG R: TCTGGTGTTAACGGGTGTGA	NM_001013611
Occludin	F: ACGGCAAAGCCAACATCTAC R: ATCCGCCACGTTCTTCAC	XM_031604121.1
JAM*-2*	F: AGACAGGAACAGGCAGTGCT R: TCCAATCCCATTTGAGGCTA	XM_031556661.1
Nutrient transporters		
GLUT1	F: ACAACACCG GCGTCATCAA R: TTGACATCAGCATGGAGTTACG	NM_205209.1
CAT1	F: ATGTAGGTTGGGATGGAGCC R: AACGAGTAAGCCAGGAGGGT	XM_015277949.1
LAT1	F: CTCTCTCTCATCATCTGGGC R: TCATTCCTGGGTCTGTTGCT	XM_415975
PepT1	F: TTTCCTTTACATCCCTCTCC R:TCACTTCTACTCTCACTC	NM-204365
House keeping		
GAPDH	F: CAACCCCCAATGTCTCTGTT R: TCAGCAGCAGCCTTCACTAC	NM205518
TBP	F: GTCCACGGTGAATCTTGGTT R: GCGCAGTAGTACGTGGTTCTC	Acc:8484

β-Defensin-1: beta defensin-1; CLDN-1: claudins-1; MUC-2: mucin-2; JAM-2: junctional adhesion molecule-2; GLUT1, glucose transporter-1; CAT1: Cationic amino acid transporter-2; LAT1: L-type amino acid transporter-1; PepT1: peptide transporter-1; GAPDH: glyceraldehyde-3-phosphate dehydrogenase; TBP: TATA-binding protein.

**Table 5:** Chemical analysis of unfermented barley (UFB) and fermented barley treated with exogenous enzymes (FBEs).

Parameter	UFB ^1^	FBEs ^2^	SEM	*p*-Value
Dry matter (%)	89.60 ^b^	90.90 ^a^	0.39	<0.030
Crude protein (%)	10.75 ^b^	11.92 ^a^	0.11	<0.001
Crude fiber (%)	5.30 ^a^	3.25 ^b^	0.39	0.040
Ether extract (%)	1.70	1.62	0.33	0.060
Lignin (%)	12.23 ^a^	8.96 ^b^	0.24	<0.001
Cellulose (%)	14.69 ^a^	10.35 ^b^	0.77	<0.001
Hemicellulose (%)	27.19 ^a^	28.22 ^b^	0.31	<0.006
Neutral detergent fiber (%)	54.11 ^a^	47.53 ^b^	0.27	<0.001
Acid detergent fiber (%)	26.96 ^a^	19.31 ^b^	0.37	0.030
Reduced sugar (g/100 g)	2.36 ^b^	8.19 ^a^	0.68	0.020
pH	6.25 ^a^	4.25 ^b^	0.15	<0.006
Total lactic acid bacteria (log CFU/g feed)	4.57 ^b^	7.96 ^a^	0.50	<0.001
*Bacillus* spp. log CFU/g feed	3.96 ^b^	6.33 ^a^	0.35	<0.001
Non-phytate phosphorus (g/kg)	2.03 ^b^	2.82 ^a^	0.16	<0.007
Phytase activity (FTU/kg)	59.36 ^b^	225.51 ^a^	0.83	<0.001

^1^ UFB (unfermented barley), ^2^ FBEs (microbially and enzymatically fermented barley). Values are specified as means ± standard error. Number of replicates analyzed = 5. CFU (colony forming unit). ^a,b^ Means within the equivalent row with dissimilar superscripts express statistical variance (*p* < 0.050).

**Table 6:** Evaluating the growth performance parameters (days 1–38) of broiler chickens fed different levels of microbially fermented barley treated with fibrolytic exogenous enzymes.

Item	Control	FBEs5%	FBEs10%	FBEs15%	SEM	*p*-Value
Starter (1–10 days)						
Initial BW	44.33	46.00	45.00	46.33	0.96	0.280
BW (g/bird)	307 ^b^	310 ^b^	335 ^a^	299 ^b^	13.36	0.020
BWG (g/bird)	262 ^b^	264 ^b^	290 ^a^	252 ^b^	14.35	0.004
FCR	1.39 ^a^	1.38 ^a^	1.24 ^b^	1.44 ^a^	0.01	0.007
FI (g/bird)	365	364	359	363	12.47	0.480
Grower (11–20 days)						
BW (g/bird)	1157 ^b^	1176 ^b^	1221 ^a^	1181 ^b^	6.30	<0.001
BWG (g/bird)	850 ^b^	865 ^ab^	886 ^a^	882 ^a^	8.40	<0.001
FCR	1.79 ^a^	1.74 ^a^	1.64 ^b^	1.67 ^b^	0.02	<0.001
FI (g/bird)	1521 ^a^	1507 ^ab^	1449 ^b^	1467 ^ab^	3.51	0.020
Finisher (21–38 days)						
BW (g/bird)	2532 ^c^	2597 ^b^	2650 ^a^	2588 ^b^	12.83	<0.001
BWG (g/bird)	1376 ^c^	1421 ^ab^	1429 ^a^	1407 ^b^	9.20	0.010
FCR	1.83 ^a^	1.75 ^b^	1.71 ^c^	1.76 ^b^	<0.001	<0.001
FI (g/bird)	2512 ^a^	2486 ^ab^	2447 ^b^	2480 ^ab^	2.60	<0.001
Overall (1–38 days)						
BWG (g/bird)	2488 ^c^	2551 ^b^	2605 ^a^	2541 ^b^	12.39	0.030
FI (g/bird)	4398 ^a^	4357 ^ab^	4255 ^c^	4301 ^bc^	11.32	0.020
FCR	1.77 ^a^	1.71 ^b^	1.63 ^c^	1.69 ^b^	0.01	<0.001

BW: body weight; BWG: body weight gain; FI: feed intake; FCR: feed conversion ratio. Control: birds fed on basal diet; FBEs5% (birds fed dietary 5% microbially fermented barley treated with exogenous enzymes), FBEs10% (birds fed dietary 10% microbially fermented barley treated with exogenous enzymes), FBEs15% (birds fed dietary 15% microbially fermented barley treated with exogenous enzymes). ^a–c^ Means within the equivalent row with dissimilar superscripts express statistical variance (*p* < 0.050).

**Table 7:** Evaluating the total tract metabolizability and digestive enzymes’ activities of broiler chickens fed with different levels of microbially fermented barley treated with exogenous enzymes.

Item	Control	FBEs5%	FBEs10%	FBEs15%	SEM	*p*-Value
Nutrients metabolizability (%)						
Dry matter	78.99 ^c^	81.78 ^b^	83.26 ^a^	82.00 ^b^	0.63	0.001
Crude protein	72.12 ^c^	74.36 ^b^	76.36 ^a^	74.12 ^b^	0.75	0.020
Crude fiber	22.99 ^c^	25.56 ^b^	27.36 ^a^	25.24 ^b^	0.83	0.030
Phosphorus	48.69 ^d^	54.66 ^c^	60.36 ^b^	65.47 ^a^	0.29	0.040
Duodenal pH	6.12 ^a^	5.64 ^b^	5.42 ^bc^	5.12 ^c^	0.13	0.030
Jejunal digesta viscosity	3.65	3.69	3.52	3.72	0.11	0.060
Digestive enzymes (U/mg protein)						
Lipase	30.93 ^b^	36.68 ^a^	37.53 ^a^	38.07 ^a^	0.44	0.001
Amylase	140.57 ^b^	145.60 ^a^	149.33 ^a^	146.27 ^a^	1.29	0.001
Trypsin	410 ^c^	469 ^b^	475 ^b^	492 ^a^	4.08	0.030
Chymotrypsin	12.80 ^c^	15.30 ^b^	16.40 ^ab^	20.91 ^a^	0.24	0.010
Maltase	153 ^c^	155 ^b^	163 ^a^	165 ^a^	1.32	0.001
Sucrase	54.10 ^c^	58.90 ^b^	60.40 ^a^	61.40 ^a^	0.0.12	0.010

Control: birds fed on basal diet; FBEs5% (birds fed dietary 5% microbially fermented barley treated with exogenous enzymes), FBEs10% (birds fed dietary 10% microbially fermented barley treated with exogenous enzymes), FBEs15% (birds fed dietary 15% microbially fermented barley treated with exogenous enzymes). ^a–d^ Means within the equivalent row with dissimilar superscripts express statistical variance (*p* < 0.050).

**Table 8:** Evaluating the biochemical and immune-related markers of broiler chickens fed with different levels of microbially fermented barley treated with exogenous enzymes.

Item	Control	FBEs5%	FBEs10%	FBEs15%	SEM	*p*-Value
IgM (ng/L)	2.10 ^b^	0.55	3.60 ^ab^	3.77 ^a^	0.55	0.020
IgG (ng/L)	1.83 ^b^	0.38	2.35 ^ab^	2.60 ^a^	0.38	0.090
Lysozyme (U/mL)	109.43 ^c^	3.30	116.73 ^ab^	118.63 ^a^	3.30	<0.001
NO (μmol/L)	4.37 ^a^	0.26	4.10 ^ab^	3.87 ^b^	0.26	<0.001
Uric acid (mg/dL)	11.70	0.11	11.20	11.43	0.11	0.060
Creatinine (mg/dL)	0.63	0.05	0.58	0.57	0.05	0.030
ALT (U/L)	11.67	0.05	11.87	11.67	0.05	0.090
AST (U/L)	56.67	5.31	56.23	57.53	5.31	0.120
Cholesterol (mg/dL)	149.17 ^a^	2.63	145.60 ^a^	140.57 ^b^	2.63	0.020
Triglycerides (mg/dL)	89.23 ^a^	4.28	88.20 ^ab^	81.63 ^b^	4.28	<0.001
HDL-c (mg/dL)	34.33 ^b^	1.82	37.53 ^ab^	38.07 ^a^	1.82	0.030
LDL-c (mg/dL)	80.90 ^a^	2.04	74.49 ^a^	66.44 ^b^	2.04	0.060

IgM: immunoglobulin M; IgG: immunoglobulin G; NO: nitric oxide; ALT: alanine transaminase; AST: aspartate transaminase; HDL-C: high-density lipoprotein cholesterol; LDL-C: low-density lipoprotein cholesterol; VLDL-C: very-low-density lipoprotein cholesterol. Control: birds fed on basal diet; FBEs5% (birds fed dietary 5% microbially fermented barley treated with exogenous enzymes), FBEs10% (birds fed dietary 10% microbially fermented barley treated with exogenous enzymes), FBEs15% (birds fed dietary 15% microbially fermented barley treated with exogenous enzymes). ^a–c^ Means within the equivalent row with dissimilar superscripts express statistical variance (*p* < 0.050).

**Table 9:** Evaluating the redox status of broiler chickens’ intestinal tissues and breast muscle fed with different levels of microbially fermented barley treated with exogenous enzymes.

Item	Control	FBEs5%	FBEs10%	FBEs15%	SEM	*p*-Value
Intestinal tissues						
GSH-Px (U/mg protein)	144.10 ^b^	151.01 ^b^	150.47 ^b^	163.01 ^a^	1.12	<0.001
SOD (U/mg protein)	20.27 ^b^	21.03 ^b^	22.06 ^ab^	23.80 ^a^	1.06	0.010
Catalase (U/mg protein)	15.80 ^c^	18.20 ^c^	25.13 ^b^	25.08 ^a^	0.93	<0.001
T-AOC (U/mg protein)	1.59 ^c^	1.56 ^c^	1.71 ^b^	1.83 ^a^	0.26	<0.001
ROS (μL/g tissue)	6.31 ^a^	5.50 ^b^	5.10 ^bc^	4.86 ^c^	0.39	0.030
H_2_O_2_ (μmoL/g tissue)	3.96 ^a^	3.60 ^b^	3.41 ^bc^	3.08 ^c^	0.11	<0.001
Malondialdehyde (nmoL/mL)	6.50 ^a^	6.07 ^a^	5.21 ^b^	4.45 ^c^	0.22	0.030
Breast muscle						
GSH-Px (U/mg protein)	152.83 ^d^	155.03 ^c^	160.07 ^b^	168.33 ^a^	0.86	0.030
SOD (U/mg protein)	36.40 ^d^	50.40 ^c^	59.43 ^b^	66.07 ^a^	0.12	<0.001
Catalase (U/mg protein)	10.17 ^d^	12.87 ^c^	13.97 ^b^	15.90 ^a^	0.21	0.030
T-AOC (U/mg protein)	1.80 ^b^	2.13 ^ab^	2.53 ^a^	2.50 ^a^	0.11	0.010
ROS	7.86 ^a^	5.63 ^b^	4.99 ^bc^	4.20 ^c^	0.09	0.230
H_2_O_2_ (μmoL/g tissue)	3.89 ^a^	3.72 ^b^	3.12 ^c^	3.10 ^c^	1.20	0.120
Malondialdehyde (nmoL/mL)	7.50 ^a^	7.30 ^ab^	6.10 ^ab^	5.37 ^b^	0.63	0.290

Total antioxidant capacity (T-AOC), superoxide dismutase (SOD), reactive oxygen species (ROS), glutathione (GSH-Px), hydrogen peroxide (H_2_O_2_). Control: birds fed on basal diet; FBEs5% (birds fed dietary 5% microbially fermented barley treated with exogenous enzymes), FBEs10% (birds fed dietary 10% microbially fermented barley treated with exogenous enzymes), FBEs15% (birds fed dietary 15% microbially fermented barley treated with exogenous enzymes). ^a–d^ Means within the equivalent row with dissimilar superscripts express statistical variance (*p* < 0.050).

**Table 10:** Evaluating the intestinal morphology of broiler chickens fed with different levels of microbially fermented barley treated with exogenous enzymes.

Item	Control	FBEs5%	FBEs10%	FBEs15%	SEM	*p*-Value
Intestinal tissues						
Villus height duodenum, µm	1166 ^c^	1229 ^c^	1245 ^a^	1233 ^a^	1.12	<0.001
Crypt depth duodenum, µm	254.33 ^d^	215.32 ^c^	182.67 ^b^	195.00 ^a^	1.06	0.010
Villus height: Crypt depth	4.58 ^d^	5.71 ^c^	6.82 ^b^	6.33 ^a^	0.93	<0.001

^a–d^ Means within the same row with dissimilar superscripts express statistical variance (*p* < 0.050). Control: birds fed on basal diet; FBEs5% (birds fed dietary 5% microbially fermented barley treated with exogenous enzymes), FBEs10% (birds fed dietary 10% microbially fermented barley treated with exogenous enzymes), FBEs15% (birds fed dietary 15% microbially fermented barley treated with exogenous enzymes).

## Data Availability

The data are accessible from the corresponding author upon request.
